# Genotyping of Environmental and Clinical *Stenotrophomonas maltophilia* Isolates and their Pathogenic Potential

**DOI:** 10.1371/journal.pone.0027615

**Published:** 2011-11-15

**Authors:** Martina Adamek, Jörg Overhage, Stephan Bathe, Josef Winter, Reinhard Fischer, Thomas Schwartz

**Affiliations:** 1 Microbiology of Natural and Technical Interfaces Department, Institute of Functional Interfaces (IFG), Karlsruhe Institute of Technology (KIT), Karlsruhe, Germany; 2 German National Academic Foundation, Bonn, Germany; 3 Department of Biology for Engineers and Biotechnology of Wastewater Treatment (IBA), Karlsruhe Institute of Technology (KIT), Karlsruhe, Germany; 4 Department of Microbiology, Institute for Applied Biosciences, Karlsruhe Institute of Technology (KIT), Karlsruhe, Germany; Indian Institute of Science, India

## Abstract

*Stenotrophomonas maltophilia* is a highly versatile species with useful biotechnological potential but also with pathogenic properties. In light of possible differences in virulence characteristics, knowledge about genomic subgroups is therefore desirable. Two different genotyping methods, rep-PCR fingerprinting and partial *gyrB* gene sequencing were used to elucidate *S. maltophilia* intraspecies diversity. Rep-PCR fingerprinting revealed the presence of 12 large subgroups, while *gyrB* gene sequencing distinguished 10 subgroups. For 8 of them, the same strain composition was shown with both typing methods. A subset of 59 isolates representative for the *gyrB* groups was further investigated with regards to their pathogenic properties in a virulence model using *Dictyostelium discoideum* and *Acanthamoeba castellanii* as host organisms. A clear tendency towards accumulation of virulent strains could be observed for one group with *A. castellanii* and for two groups with *D. discoideum*. Several virulent strains did not cluster in any of the genetic groups, while other groups displayed no virulence properties at all. The amoeba pathogenicity model proved suitable in showing differences in *S. maltophilia* virulence. However, the model is still not sufficient to completely elucidate virulence as critical for a human host, since several strains involved in human infections did not show any virulence against amoeba.

## Introduction

The genus *Stenotrophomonas* belongs, together with *Xanthomonas*, to the γ-β subclass of proteobacteria [1]. The first detected *S. maltophilia* species was initially described as *Pseudomonas maltophilia* in 1981, later reclassified as *Xanthomonas maltophilia* and finally classified as *Stenotrophomonas maltophilia* [2], [3], [4]. In the following years, additional species such as *S. nitritireducens* [5], *S. rhizophila* [6], *S. acidaminiphila* [7], *S. koreensis* [8], *S. terrae* and *S. humi* [9], *S. chelatiphaga* [10], *S. ginsengisoli* [11], *S. daejonensis* [12] as well as *S. panachihumi* [13], and *S. pavanii* [14] were described. *S. dokdonensis* was recently moved to the genus *Pseudoxanthomonas* [15], [16]. *S. africana*, which had been introduced as a new species in 1997, was later found to be synonymous to *S. maltophilia* [17], [18].

*Stenotrophomonas* spp. and especially the human opportunistic pathogen *S. maltophilia* were found to occur ubiquitously in natural and anthropogenic environments [19]. A number of investigations concentrated on the genetic characteristics of different selections of isolates [20], [21], [22], [23], [24]. Other studies focused on the genetic and phenotypic properties of clinical *Stenotrophomonas* isolates obtained over a certain time period in a single, or some selected hospitals [25], [26], [27]. These publications revealed a high genetic and phenotypic diversity on the genus and species levels and suggested that some phylogenetic groups may have increased potential to cause infections compared to others. A significant difference between mutation frequencies of clinical and environmental *S. maltophilia* strains was revealed in a study by Turrientes *et al.* [28]. Clinical strains were mostly hypermutator strains, due to the impairment of the *mutS* gene.

Clinical manifestation of *S. maltophilia* infections results, in most documented cases, in pneumonia, blood-stream, wound and urinary-tract infections [29]. *S. maltophilia* isolates have been described to enhance inflammatory response, such as interleukin-8-expression in airway epithelial cells and TNF-(tumor necrosis factor)-α expression in macrophages, which can contribute to airway inflammation. Excessive inflammatory response to *S. maltophilia* strains isolated from cystic fibrosis (CF) patients was detected during a murine lung infection model [30]. Cytotoxic effects against Vero, HeLa and HEp-2 cell lines were characterized for five of ten tested clinical *S. maltophilia* strains [31]. Virulence for strain K279a was shown in a nematode model with *Caenorhabditis elegans* N2 [32].

A polyphasic taxonomic approach identified 10 genomic groups within the genus by amplified fragment length polymorphism (AFLP) analysis [21]. These groupings were reproduced partly by a restriction fragment length polymorphism (RFLP) analysis of the *gyrB* gene [23]. On a 16S rRNA gene level, *S. maltophilia* strains were found to form two clusters. The larger one was comprised of strains from clinical and environmental origin; a smaller separate 16S rRNA lineage also was identified by Minkwitz and Berg (2001) as group E2 [22]. It should be noted that this lineage contains only environmental isolates. Recent investigations of *S. maltophilia* isolates by multilocus sequence typing (MLST) and MALDI-TOF mass spectrometry, confirmed the occurrence of nine genogroups as described by AFLP and suggested the presence of five additional groups [33], [34]. *S. maltophilia* species contain pathogenic isolates and strains of biotechnological potential, such as xenobiotic degraders or strains with biocontrol properties, which may or may not have pathogenic properties. Hence, an in-depth knowledge about the existence of genomic subgroups, differences in their pathogenicity, and the development of diagnostic tools to differentiate between these groups is desirable.

This collection was analyzed by rep (repetitive extragenic palindromic)-PCR, which has proven to be a powerful tool for molecular diagnostics purposes. This method makes use of the fact that microbial genomes contain a variety of repetitive sequences (up to 5% of the genome). Although their function has mostly not been elucidated so far, they may serve as a basis for the creation of specific genetic fingerprints for bacterial strains with a resolution beyond the species level [35]. In this way, DNA sequences between the repetitive parts are amplified. The result is a mixture of PCR products of different length displaying unique, strain-specific fingerprints when separated by electrophoresis. Additionally, sequences of housekeeping genes, such as the *gyrB* gene that encodes DNA-gyrase, display a higher phylogenetic resolution than the 16S rRNA gene. Consequently, it should be possible to distinguish among genetic groupings beyond the species level.

A simple model for studying host-pathogen interactions can be provided by the free-living social amoeba *Dictyostelium discoideum*. Its usual habitat is the soil, where the amoeba predates other microorganisms, including bacteria. It is supposed that bacterial virulence mechanisms have been developed as a defense to withstand predation by amoebae. *D. discoideum* has been successfully used to elucidate infection pathways for *Pseudomonas aeruginosa* [36], [37], *Aeromonas* species [38], *Vibrio cholerae* [42], and others [43]. *Acanthamoeba castellanii* is a free living amoeba, commonly found in soil and water. As an opportunistic pathogenic organism it is involved in keratitis, and in rare cases, within immunocompromised patients in meningitis. *Acanthamoeba* species were likely used as model organism to elucidate bacterial virulence for *P. aeruginosa* [44] and *Mycobacterium kansasii* [45]. *Acanthamoebae* have the advantage to grow at temperatures between 30–35°C, making them useful hosts to study bacterial pathogenicity at temperatures similar to the human host. A good correlation for virulence observations in amoeba compared to mammalian models has been reported previously [37].

The study reported here was aimed at investigating *S. maltophilia* genetic diversity in the light of the current data on the intra- and interspecies diversity within the genus. These data were then used to decide on a subset of strains for comparison of virulence potential expressed in an amoeba model of infection. The strain collection investigated in this study comprised 106 recently isolated strains, 49 of them were of clinical and 57 of environmental origin, emphasizing an equal ratio of clinical to environmental strains. A collection of 62 reference strains of clinical and environmental origins described in previous studies was additionally included for comparative purposes.

## Materials and Methods

### Origin of Environmental and Clinical Bacterial Strains

Bacterial strains used in this study can be retaliated in table S1 of the supplementary material. Of these, 57 different uncharacterized *S. maltophilia* isolates were newly isolated from the environment: 18 were isolated from freshwater sediments taken from a branch of the river Rhine near Karlsruhe, and 3 isolates from tap water sampled at the municipal hospital of Karlsruhe. An amount of 27 isolates originated from activated sludge and 9 from the sewage plant effluent of different wastewater treatment plants, and were therefore referred as anthropogenic influenced environmental isolates.

Twenty strains were obtained either from the German Collection of Microorganisms and Cell Cultures (DSMZ), or the Belgian Coordinated Culture Collection at the Laboratory for Microbiology at the University of Ghent (LMG). 61 isolates were obtained recently from strain collections of the Municipal Hospital in Karlsruhe (Germany) and the University Hospital in Freiburg (Germany). Furthermore, 42 isolates were obtained from the University of Rostock (Germany) and one from Dr. Daniel van der Lelie (Brookhaven National Laboratory), one from Dr. Max Dow (University College Cork, Ireland) as well as one from Prof. Ake Hagström (University of Kalmaer, Sweden).

*Klebsiella aerogenes* DSM 2026 and *Pseudomonas aeruginosa* PA 14 were used as positive and negative control respectively for growth of amoeba in the virulence assay.

### Media for Isolation or Selective Enrichment of *S. maltophilia* and Culture Conditions

*Stenotrophomonas maltophilia* strains were isolated from environmental samples using two different approaches: (i) Serial dilutions of aquatic samples or soil extracts were directly plated on *Xanthomonas maltophilia*-selective agar medium (XMSM) according to Juhnke & des Jardin [46]. Colonies grown after 3 days of incubation at 30°C were considered to be *S. maltophilia*. (ii) Environmental samples were incubated overnight in nutrient broth [5 g/l peptone, 3 g/l meat extract, 50 g/l NaCl (all supplied from Merck, Darmstadt, Germany)], containing 0.5 mg/ml methionine (Sigma-Aldrich, Taufkirchen, Germany) in order to select methionine-auxotrophic bacteria, including *S. maltophilia*. Aliquots of these cultures were plated on Mueller-Hinton agar (Merck) and Imipenem disks (Beckton Dickinson, Heidelberg, Germany) were applied on the bacterial lawn. Tiny colonies were observed in the inhibition zone after 18 hours of growth at 30°C [47]. To determine which of these colonies were *S. maltophilia*, all candidate isolates were further investigated by PCR with *S. maltophilia*-specific primers SM1f/SM4, as will be described later, to confirm their taxonomic status.

### PCR

Heat denatured cell extracts were used as template DNA in all PCR reactions. All PCR primers were obtained from MWG Operon (Darmstadt, Germany). PCR-reaction supplements were provided by Fermentas (St. Leon-Rot, Germany).

*Stenotrophomonas maltophilia* strains were screened using a specific PCR reaction to confirm taxonomic identities. The primers used were SM1f (5′-GTT GGG AAA GAA ATC CAG C-3′) targeting the 16S rRNA gene (position 441) and SM4 (5′-TTA AGC TTG CCA CGA ACA G-3′) targeting the 23S rRNA gene (position 594) [48]. PCR was conducted in 25 µl reaction volumes containing 0.5 units of polymerase (Fermentas TrueStart Taq DNA polymerase) in 1× reaction buffer, 2.5 mM MgCl_2_, 0.2 mM of each dNTP, 0.2 µM of each primer, and 1 µl of cell extract. Thermal cycling was conducted with an initial step of 95°C for 5 min, followed by 35 cycles of 95°C for 30 s, 55°C for 1 min, and 72°C for 2 min each. The program was concluded by an extension at 72°C for 5 min.

Additionally, all *S. maltophilia* strains were screened with primers specific to the 16S rRNA gene subgroup E2. The newly designed primers used were SMgroupE2-for (5′-TGC AGT GGA AAC TGG ACA-3′) and SMgroupE2-rev (5′-CCA TGG ATG TTC CTC CC-3′). The PCR reaction mix was the same as described above, with the thermal program consisting of an initial step of 95°C for 5 min, followed by 30 cycles of 95°C, 52°C, and 72°C for 30 s each. The program was concluded by a final extension at 72°C for 5 min.

Rep-PCR analyses were conducted with the single primers BoxA1R (5′-CTA CGG CAA GGC GAC GCT GAC G-3′) and (GTG)_5_ (5′-GTG GTG GTG GTG GTG-3′) according to Versalovic *et al.* [49]. The PCR reaction mix consisted of 25 µl total volume containing 1 unit of Taq polymerase in 1× reaction buffer, 10% DMSO, 5 mM MgCl_2_, 1 mM of each dNTP, 5 µM of primer, and 1 µl of cell extract. Thermal cycling was conducted with an initial denaturation at 95°C for 10 min, followed by 25 cycles of 95°C for 45 s, 50°C (primer BoxA1R) or 47°C (primer (GTG)_5_) for 1.5 min, 65°C for 8 min each, and concluded by a final extension of 65°C for 16 min [49].

Partial *gyrB* gene sequences were amplified using universal primers UP-1 and UP-2r and specific primers UP-1S and UP-2S as described by Yamamoto and Harayama [50]. Amplicons were custom-sequenced using UP-1S as sequencing primer.

PCR products were separated by electrophoresis in an agarose gel. DNA patterns were visualized by staining with 2 µg/ml ethidium bromide (Merck). Of all strains isolated at the same time from the same origin and showing identical rep-PCR profiles, only one representative strain was chosen for analysis to avoid clonally identical isolates.

### Phylogenetic Analyses

Rep-PCR profiles were analyzed by BioNumerics 5.0 using Pearson's correlation coefficient with UPGMA (Unweight Pair Group Method with Arithmetic Mean) clustering of averaged profile similarities. Profiles with similarity values higher than 45% were condensed into clusters and clusters containing at least three isolates were considered to form a genogroup. Cluster analyses were carried out using MEGA4 after alignment with ClustalW. Phylogenetic trees were constructed using the neighbour-joining algorithm with 1000 bootstrap repetitions.

### Virulence assays

In order to assess the virulence characteristics of *S. maltophilia*, we used an adjusted version of the plate killing assay described for *P. aeruginosa* [36]. Amoeba used for this assay were the axenic *Dictyostelium discoideum* strain Ax2, and *Acanthamoeba castellanii* strain ATTC 30234. *D. discoideum* cells were grown in HL5 Medium, as described by Fey *et al.* [51]. *A. castellanii* was grown in PYG-medium, as described by Rowbotham [52]. Cells were incubated in cell culture flasks (Greiner Bio One, Frickenhausen, Germany) at 22.5°C for *D. discoideum* and at 30°C for *A. castellanii* and subcultured twice a week.

The bacterial strains used for pathogenicity testing are indicated in table S1 from the supplementary material. The optical density at 600 nm (OD_600_) of a bacterial overnight culture was adjusted to 1.6–1.8 by dilution in LB (Luria-Bertani) broth (Becton Dickinson). For co-incubation of bacteria with amoebae, M9-agar plates were used (6 g Na_2_HPO_4_, 3 g KH_2_PO_4_, 0.5 g NaCl, 1 g NH_4_Cl, 10 ml 100 mM MgSO_4_, 10 ml 10 mM CaCl_2_, 10 ml 2 M glucose (all Merck), 10 ml methionine (5 mg/ml) (Sigma-Aldrich), and 20 g agar (Merck) per liter). 1 ml of the bacterial suspension was plated on a M9-Agar plate. Plates were dried under a laminar flow bench for about three hours to obtain a dry and even bacterial lawn.

Amoeba grown for 2–4 days were harvested by centrifugation at 1,600× g for 1 minute, washed once in PBS-buffer (8.18 g NaCl, 1.8 g Na_2_HPO_4_, 0.2 g KCl, 0.24 g KH_2_PO_4_ (all Merck) per litre), and resuspended in PBS buffer. Amoebae were chilled on ice for 15 minutes and the number of cells was adjusted to about 2×10^6^ cells per ml. This solution was diluted 1∶1 until a final density of 1000 amoebae per ml was achieved. From each of these solutions 5 µl droplets, containing 10,000, 5,000, 2,500 up to 5 amoebae were spotted on the bacterial lawn. The plates were incubated at 30°C for 3 days with *A. castellanii* and at 22.5°C for 5 days with *D. discoideum*. The highest dilution, at which growth of the amoebae in form of a plaque was visible, was reported. Experiments were carried out at least in triplicate, with *P. aeruginosa* and *Klebsiella aerogenes* as positive and negative control for each single approach, to ensure reproducibility of the results.

## Results

### Taxonomic kinship of *S. maltophilia* isolates

Two different discriminative methods were used to give insight into the genetic diversity. In the following attempt we characterized *S. maltophilia* genetic diversity by different methods: (i) rep-PCR, a method highly sensitive to genetic differences on a genome wide level, and (ii) *gyrB* gene sequencing, characterizing differences in one single housekeeping gene reflecting intraspecies diversity.

### Rep-PCR Fingerprinting

**Figure 1:**
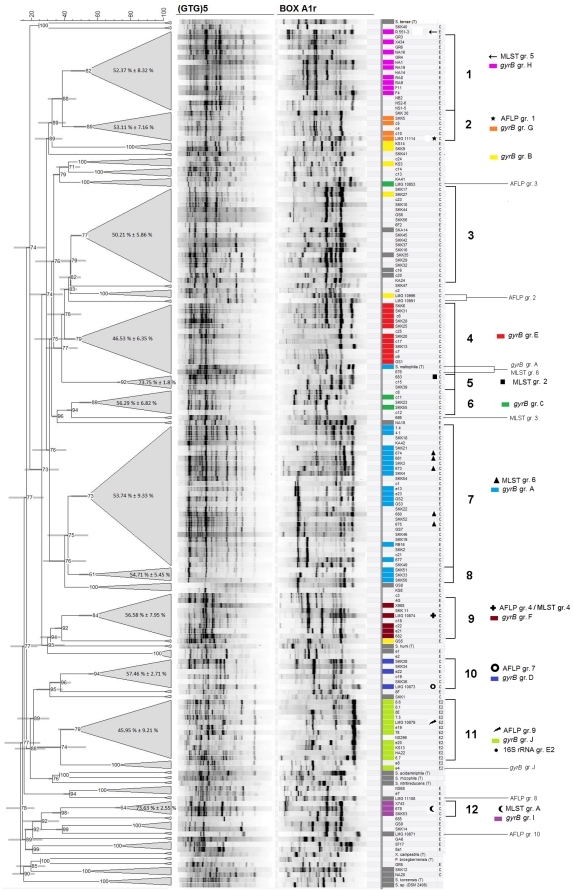
Dendrogram based on (GTG)5 and BoxA1R fingerprint profile similarities. Analysis was performed by using Pearson's correlation with UPGMA clustering. Cophenetic correlation values are shown for each branch. Grey bars at the branches are representing the error bars. In each condensed cluster assigned as genetic grouop similarity values (%) ± standard deviation (%) are shown. Reference strains from previous AFLP/MLST groupings were highlighted with specific symbols. Reference isolates accounted to 16S rRNA groups E2 were shown with a black point. Isolates characterized additionally by *gyrB* gene sequencing (Figure 2) were marked with coloured boxes representing the *gyrB* genetic group. Strains belonging to no *gyrB* group were highlighted in grey.

To evaluate the genetic diversity for a large subset of *S. maltophilia* isolates, rep-PCR fingerprinting using two different primers (BoxA1R and (GTG)_5_) was performed. Altogether 171 bacterial isolates, including *Stenotrophomonas* species type strains, *Xanthomonas campestris* pv. *campestris*, and *Pseudoxanthomonas broegbernensis* were characterized. To avoid clonality, isolates from the same site, showing identical fingerprints were removed from the study. Results are shown in form of a dendrogram in figure 1, which was constructed by comparing combined profile similarities of both fingerprint types, using Pearson's correlation with UPGMA clustering. It was possible to distinguish isolate groups due to the presence of distinct signature bands, which appeared only within certain clusters. For example, characteristic bands can be seen in the BoxAR1 profile of group 3 or group 11 and in the (GTG)_5_-profiles of group 7. We observed that these clusters shared at least an average similarity of 45%. Therefore we defined a threshold value of 45% profile similarity, and a minimum of three strains for the definition of a cluster. Under these premises twelve groups (delineated as 1–12 in figure 1 on the right side) were determined. On the left-hand side of figure 1 average similarity values including the standard deviations for the single clusters are presented. Grey bars are representing the error bars, with the cophenetic correlation coefficient assigned for each branch.

To get an overview of the distribution of clinical and environmental isolates, we described just the tendencies we noticed, for example when many strains from a similar isolation source clustered in one group, or some groups were comprised of only clinical or of only environmental isolates. Any further relevant details (as far as known) can be seen in table S1 in the supplementary material. Of the twelve described groups, most were comprised of both clinical and environmental isolates. The largest of them was cluster 7 with 28 isolates. Here a tendency towards an accumulation of respiratory tract isolates, 13 of 19 clinical isolates, could be observed. For the environmental isolates in this group it should be noted that five of nine originated from wastewaters. Clusters 3 and 4 contained both, clinical and environmental isolates; the majority of them were of clinical origin. While cluster 3 showed a higher number of respiratory tract isolates, cluster 4 exhibited no tendency for a specific isolation site. Furthermore, three smaller clusters, namely 9, 10, and 12, were comprised of clinical and environmental strains. The two large clusters 1 and 11 were found to contain just environmental strains. Cluster 1 showed a very large number of wastewater isolates (12 of 16), whereas in cluster 11 most isolates were from freshwater sediment. Finally, there were four small groups (2, 5, 6, and 8) containing only clinical isolates.

Twelve further clusters containing only two isolates could be identified. However, we focused on the twelve larger groups. Thirteen *S. maltophilia* isolates did not cluster at all. *X. campestris* pv. *campestris*, *P. broegbernensis*, and the *Stenotrophomonas* spp. type strains could not be distinguished from other *S. maltophilia* isolates by this method.

### *gyrB* Gene Sequence Analysis

Partial *gyrB* gene sequences (about 500 to 700 bp of the 5′ end) were obtained for 98 *S. maltophilia* strains, including the type strain, six additional *Stenotrophomonas spp.* type strains, and four strains of *S. rhizophila*. This experiment was conducted in parallel to the rep-PCR fingerprint analysis. The favored strains were newly isolated strains from freshwater and wastewater samples, as well as the hospital samples from Karlsruhe and Freiburg. As reference strains, we used representative strains for each genetic group from the study of Hauben *et al.* [21] (LMG) ten clinical, and ten environmental strains from the study of Minkwitz *et al.* [22].

**Figure 2:**
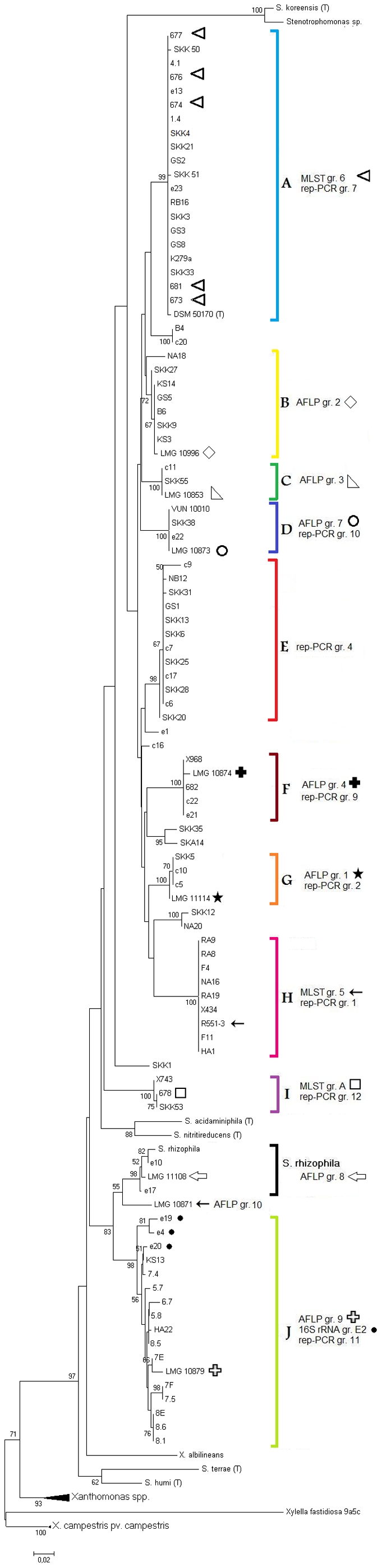
Neighbour-joining tree based on partial *gyrB* gene sequences. Neighbour-joining tree of *Stenotrophomonas* strains with *Xanthomonads* and *Xylella fastidiosa* used as outgroups. Reference strains from previous AFLP/MLST groupings were highlighted with specific symbols. Reference isolates accounted to 16S rRNA groups E2 were shown with a black point.

A neighbour-joining analysis revealed a phylogenetic tree with eleven clusters of three and more *S. maltophilia* isolates (Figure 2). To distinguish them from rep-PCR clusters they were named A–J. One of these clusters contained three *S. rhizophila* strains, including the species type strain, and was therefore named *S. rhizophila*. Three clusters containing only two isolates could be observed. Four isolates did not group with any of the others.

Cluster A, with 22 isolates, was the largest group revealed here. It was comprised of 14 clinical and 8 environmental isolates and showed a tendency towards accumulation of bacteria from the respiratory tract (12 of 14 clinical isolates). Of the environmental isolates in this cluster, four were obtained from wastewater. Group E contained 12 isolates, ten of them were isolated from patients and only two from wastewater. While group A seemed to accumulate respiratory tract isolates, group E revealed just one respiratory isolate clustered with mostly isolates from urine samples, one from a venous catheter, one from a wound infection and four further uncharacterized human isolates. Two smaller clusters (C and G) contained only isolates of clinical origin and we found two larger clusters (H and J) that contained only environmental isolates. Whereas cluster H showed mostly wastewater isolates (7 of 9), cluster J accumulated strains isolated from freshwater sediment. In clusters B, D, F, and I the distribution of clinical and environmental isolates was quite equal. It should be noted for cluster B that all environmental isolates were isolated from wastewater, while for the other groups no obvious tendency to a certain sampling site could be documented. *S. koreensis* clustered with the *Stenotrophomonas* sp. strain 2408. Type strains of the other *Stenotrophomonas* species, *Xanthomonads*, and *Xylella fastidiosa*, which were used as outgroups, could be clearly distinguished from *S. maltophilia* isolates.

### Comparison of rep-PCR fingerprinting and *gyrB* gene sequencing results

When comparing the results of both typing methods, it is remarkable that most rep-PCR groups are reflected by *gyrB* clustering. In detail, all isolates from *gyrB* group H clustered in rep-PCR group 1, all isolates from group G clustered in group 2. Rep-group 3 strains did not cluster according to *gyrB* gene sequences. Group 4 isolates were mostly found in *gyrB* group E, with the exception of one isolate, the *S. maltophilia* type strain DSM 50170, clustering with *gyrB* group A. Group 6 isolates clustered in *gyrB* group C. In rep-PCR group 7, the largest cluster, most isolates from *gyrB* group A were found. In only a short distance from cluster 7, cluster 8 also contained isolates from *gyrB* group A. Rep-clusters 9 and 10 were in congruence with *gyrB* clusters F and D, respectively. The environmental cluster 11 contained all *gyrB* group J isolates, except one strain (e4) that grouped in direct neighbourhood with 38% sequence similarity. Finally, all strains from rep-group 12 were assembled in *gyrB* group I.

### Comparison of *S. maltophilia* groups with previous classifications of subgroups

When comparing the distribution of reference isolates included in this study, a high congruence of rep-PCR groups with AFLP [21] and MLST [33] groups could be observed. For *gyrB* sequences, all representative isolates from the previously defined groups, Hauben 1–9 and Kaiser A–E, could be retrieved in the *gyrB* groups. Reference isolates from MLST or AFLP studies are marked with different symbols in figures 1 and 2. A high correlation could be seen for reference isolates from MLST group 6, which all clustered in *gyrB* group A. Most of them could also be correlated with rep-group 7, although strain DSM 50170, the *S. maltophilia* type strain, and strain 676 belonged to rep-group 4. In *gyrB* groups B, C, D, F, G, H, I, and J respectively, reference isolates from AFLP/MLST groups 2, 3, 7, 4, 1, 5, A, and 9 could be found. While *gyrB*-groups were mostly showing the same composition as rep-PCR groups, they also correlated with MLST groups. Exceptions were MLST/AFLP group 3 strains, which were not clustering with any other *S. maltophilia* isolates in figure 1, and group 2 strains, which were only clustering with each other.

### Phenotypic classification of potential virulence of *S. maltophilia* isolates

**Figure 3:**
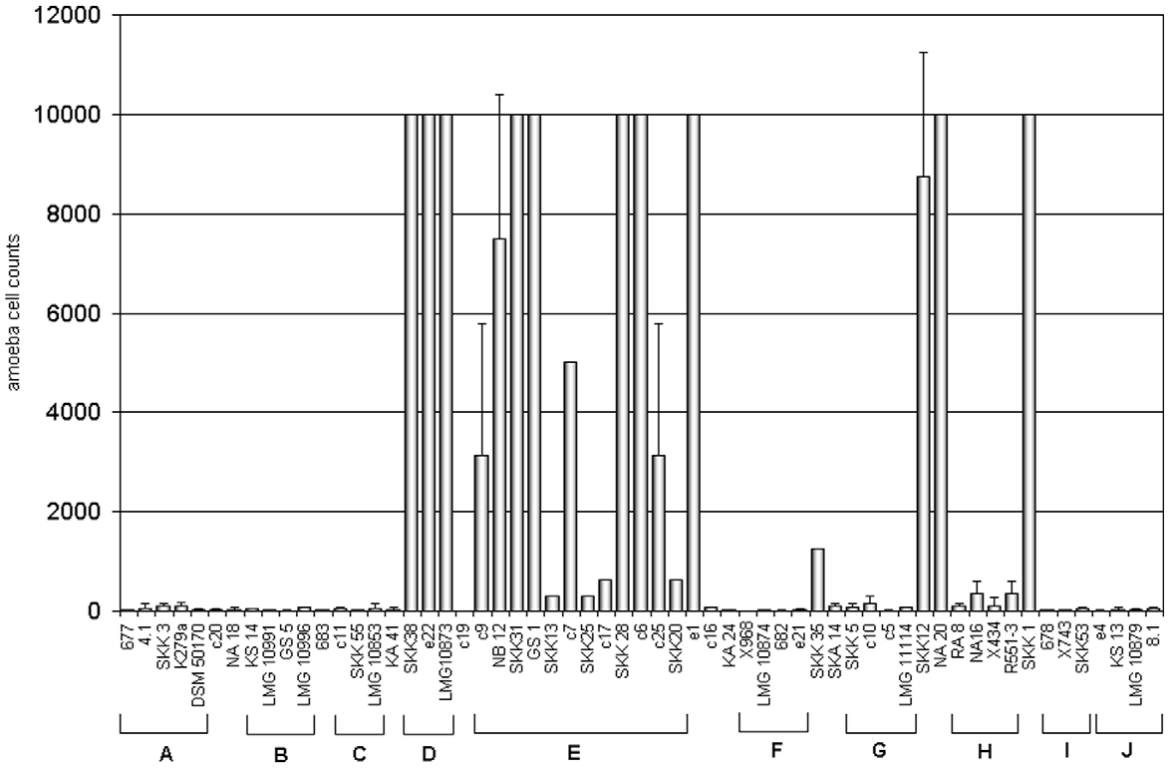
*Dictyostelium discoideum* plate killing assay. Bars are representing the number of amoeba necessary to form a plaque on the bacterial lawn of the 59 different isolates. The mean of at least three experiments was determined for each strain. Standard deviation is indicated with error bars. Genetic groups as determined by *gyrB* gene sequencing are pointed out as A–J.

**Figure 4:**
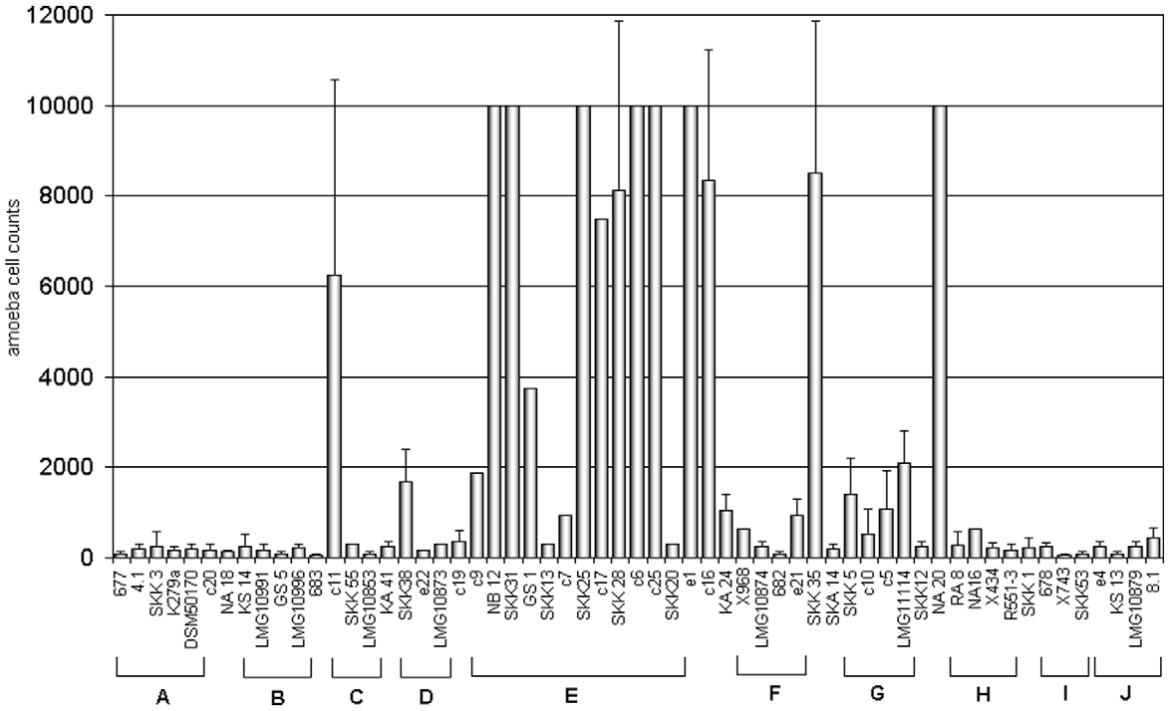
*Acanthamoeba castellanii* plate killing assay. Bars are representing the number of amoeba necessary to form a plaque on the bacterial lawn of the 59 different isolates. The mean of at least three experiments was determined for each strain. Standard deviation is indicated with error bars. Genetic groups as determined by *gyrB* gene sequencing are pointed out as A–J.

In order to get an overview of possible differences in virulence, two amoebae were used as model organisms to test a selected subset of *S. maltophilia* isolates. 59 isolates representative for the genetic groups determined via rep-PCR and *gyrB* gene sequencing were studied. Of each rep-PCR/*gyrB*-group four strains were chosen. When possible, two of them were of clinical and another two of environmental origin. From group E, first two clinical and two environmental strains were chosen. As these showed a very high virulence potential, additional eight strains from this group were tested to evaluate the thesis that isolates of this group have an increased virulence potential compared to the others. *D. discoideum* and *A. castellanii* were tested separately and spotted in different concentrations on the bacterial lawn. Dilutions with cell numbers from 10,000 to 5 amoebae were used. The number of amoeba necessary to graze a plaque into the bacterial lawn was documented as the count for the virulence potential. The more amoeba that were needed for plaque formation, the more virulent the strain was rated. Figures 3 and 4 show *S. maltophilia* isolates tested for virulence with *D. discoideum* and *A. castellanii*.

For characterization of virulence three categories were chosen. Isolates with less than 400 amoebae necessary to form a plaque were considered as non-virulent. This is consistent with the observations we made for the non-pathogenic *Klebsiella aerogenes* which we used as positive control for amoeba growth. From 400 to 2,500 amoeba used for plaque formation, isolates were seen as low-virulent. Here, a differentiation to the non virulent isolates became obvious, but was not as clear as for the isolates characterized as virulent with more than 2,500 amoeba necessary. Most *S. maltophilia* isolates showed no virulence to *D. discoideum* (Figure 3), less than 400 amoebae for plaque formation were needed for 40 isolates. Three isolates showed low virulence in a range of 400–2,500 amoebae. 16 isolates were characterized as virulent. The virulence properties were also compared with the previously defined genetic groups of the bacteria. Of the 20 virulent or low-virulent isolates, three clustered in group D and twelve in group E. The other five virulent strains e1, c16, SKK35, SKK12, NA20, and SKK1 did not cluster in any of the groups. When looking at the isolation source of the virulent strains it could be seen that some clinical and some environmental isolates were expressing virulence properties.

For *A. castellanii* (Figure 4) usually a higher concentration of amoeba was needed for plaque formation. Despite that, strains could be characterized in the same three categories as tested with *D. discoideum*. Thirty-four strains were non-virulent, 12 low-virulent, and 13 strains were classified as highly virulent. Comparing these results to the *gyrB* groups revealed that groups A, B, and I consisted only of non-virulent isolates. The majority of pathogenic strains were clustering in group E. Groups C, D, H, and J contained each one low virulent or virulent isolate next to non virulent ones. In group F, two of four isolates were low-virulent and for group G all four isolates showed low virulence properties. Again, virulence was observed for clinical and environmental strains.

Comparing results for both amoebae it could be seen that in both assays the majority of virulent strains were clustering in group E, while groups A and B were not showing virulence properties at all. Contrary to the *Dictyostelium* results for group D, virulence of all group D isolates with *A. castellanii* was lower or strains were non-virulent. Furthermore, for some single strains different virulence properties in the *Dictyostelium* approach than in the *Acanthamoeba* approach were observed. For example isolates c11, SKK25, and SKK12 showed a high virulence potential for *A. castellanii*, but no virulence for *D. discoideum*. The strain SKK35 was highly virulent for *A. castellanii*, but showed lower virulence for *D. discoideum*. All strains from *gyrB* group G were low virulent for *A. castellanii* and not virulent for *Dictyostelium*. On the other side, strains from *gyrB* group D and SKK1 were showing increased virulence for *D. discoideum*, but not for *A. castellanii*.

## Discussion

*Stenotrophomonas maltophilia* isolates can be found in a number of different surroundings, mainly in the soil, plant rhizosphere, surface water, and wastewater; but also in food, drinking water or contaminated medical care fluids [29], [19]. A major problem is its appearance as an opportunistic pathogen in hospital acquired infections. *S. maltophilia* strains isolated from infected patients are not specifically colonizing a certain tissue type, infections are quite various and most seem to depend on the patients' previous condition, treatment, and the possibility to enter the human host, for example by indwelling devices [53], [54], [55]. Community acquired infections are rare but documented [56]. Patient to patient spread is uncommon. Diversity studies on outbreaks in the same hospital revealed for most instances taxonomically different isolates [57], [26], [25].

This study tries to give an overview on a broad spectrum of *S. maltophilia* isolates from different sources. While most studies focused on clinical specimen, using environmental strains mostly as references, in this approach we decided to focus on clinical and environmental isolates not previously characterized, in equal amounts. In addition, reference isolates from previous taxonomic studies were included. One of these previous approaches was AFLP fingerprinting used to study genetic diversity of a set of 108 clinical and environmental *S. maltophilia* strains by Hauben *et al.* [21]. Overall they were able to define 10 groups of genomically related strains with a mean internal correlation of 40% similarity, which could be retrieved by DNA-DNA hybridization experiments and partly by 16S rRNA sequencing [21]. We included one strain of each of these genetic groups in our study. In the same year another study aimed for revealing genotypic and phenotypic relationships of 40 clinical and environmental *S. maltophilia* strains with regard to their isolation source [20]. They used BOX fingerprinting TGGE and PFGE after digestion with *Dra*I for genotyping and compared these data to the metabolic profiles and antibiotic resistance profiles of the strains. Although these methods proved as suitable for molecular typing, no grouping ascribable to their isolation source was possible. We also included 18 strains as reference. A recent approach developed a MLST scheme for *S. maltophilia* [33]. They included reference strains from the AFLP study and were able to confirm the presence of the same ten genetic subgroups, and described five previously uncharacterized groups. Furthermore, they were able to confirm these results by MALDI-TOF mass spectra on a subset of their strains [34]. Both approaches revealed the presence of three groups containing only, or predominantly, isolates of environmental origin (namely groups 5, 8, and 9). These results suggested that some subgroups from certain ecological origins may exhibit differences concerning their clinical relevance. We were able to include reference strains isolated from cystic fibrosis patients used in that study. Finally, we included three strains (K279a, R551-3, and SKA14) for which the complete or the draft genome sequence was available [58], [59], [60]. As new *S. maltophilia* isolates we used bacterial strains collected over a one-year period in the municipal hospital in Karlsruhe. We got 56 (SKK 1-56) strains isolated from patients and three strains isolated from tap water (x968, x743, and x434) in the hospital. Furthermore, we got 18 strains isolated from different surface water sources of Karlsruhe and 17 from wastewater treatment plants around Karlsruhe as representation for environmental distribution, and anthropogenically influenced strains present in the environment.

In our study two different methods, rep-PCR fingerprinting and *gyrB* gene sequencing, for genetic differentiation of *S. maltophilia* were tested. Thereby, not only a high similarity between groups obtained by both methods, but also a resemblance to genetic groups previously described could be seen. This proved that both methods were suitable to elucidate *S. maltophilia* intraspecies diversity. Rep-PCR fingerprinting was the method with the best time and cost efficiency and *gyrB* gene sequencing had the best results related distinguishing *Stenotrophomonas* on species and intraspecies level. To facilitate the discussion, since both methods revealed almost the same groups, we focused on *gyrB* groups. Genetic groups will be described as *gyrB* groups A–J, when not mentioned explicitly. Regarding the internal composition of the genetic groups, it was noticed that some clusters contained only clinical isolates (C and G), some only environmental isolates (H and J), and most were composed of both types. A problem for the elucidation of the internal group structure, meaning if a certain tissue or habitat specificity occurs, is that for a clear description of some groups there were still too few isolates. With only three isolates it is problematic to make assumptions. Besides, for some isolates (c1–c25) only the description “human” was known. This should be kept in mind when discussing such things as tissue specificity for some groups. As a tendency to certain tissue specificity it could be seen that in group A mostly respiratory tract strains were found. This goes along with the observation by Kaiser *et al.* [33] that most isolates taken from CF patients were clustering in MLST group 6. A predisposition of this subgroup for a facilitated inheritance of the respiratory tract could be anticipated. Because all reference isolates from the clinic of Freiburg were from CF patients and therefore mostly from MLST group 6, a possible sampling bias should be considered. But, it could be demonstrated that most respiratory tract isolates from the clinic of Karlsruhe were also found in group A, which again supports the thesis of a respiratory tract genotype. For group E mainly urinary tract isolates along with a blood culture sample and a wound isolate were found next to only one respiratory tract isolate. This raises the question if a different tissue preference by this genetic subgroup is possible.

An increased amount of wastewater isolates was seen for group H (group 1 for rep-PCR grouping). These isolates have been characterized as anthropogenically influenced environmental isolates, as bacteria might be excreted by humans or animals, become part of the wastewater treatment system and be released into the environment. While the biological step in wastewater treatment plants is designed to promote bacterial growth, accumulation of heavy metals, anti-microbial agents, and detergents was proposed for this environment as well. The load of antimicrobial agents in wastewater is correlated with the sources of the influent, as hospital wastewater or animal farming, and seasonal aspects, as a higher antibiotic consumption in winter [39]. A high selection pressure under these conditions could lead to a selection of multiresistant bacteria. Furthermore, these substances were described to enhance the co-resistance or cross-resistance to antibiotics [40]. As *S. maltophilia* is already known to harbor multiple resistances [58], possibly, group H isolates have the potential to adapt to such an environment and acquire resistance to antibiotics and/or heavy metals compared to isolates from other groups.

Regarding the group composition, it could be assumed that the groups containing only environmental isolates would show less virulence than the groups containing clinical strains. This was already shown by Pompilio *et al.* [41] in a DAB/2 mouse model. They compared two strains and showed that the dissemination and murine immune response were significantly higher for a clinical strain compared to an environmental strain, although both led to a similar mortality rate. We used an amoeba pathogenicity model to elucidate the relationship between genetic groups, sampling origin, and virulence properties.

In the recent years amoebae were used at an increasing rate as host organisms to unravel virulence pathways of bacterial pathogens. For example, an assay with *D. discoideum* was used to show that *P. aeruginosa* virulence is mediated by a type III secretion system injecting the cytotoxin ExoU [36]. Later it was observed in a *Dictyostelium* model that the *rhl* quorum sensing system of *P. aeruginosa* also plays a central role in virulence [37]. *Acanthamoeba castellanii* was successfully introduced to determine virulence of *Mycobacterium kansasii*. Results correlated with clinical virulence and genetic subtypes [43]. Host-pathogen interactions for numerous bacterial species to amoebae are described in a review by Greub and Raoult [43]. Virulence of *S. maltophilia* strain D457 has been observed in co-cultures with *D. discoideum*. A mutant of this strain (D457R) overexpressing the multidrug efflux pump SmeDEF was reported as less virulent than the wild type strain [61]. In a study on the effect of bacteria on survival and growth of *A. castellanii*, *S. maltophilia* ATCC 420207 was described as good food source for *A. castellanii* [62].

In our study we were able to show differences in virulence properties of *S. maltophilia* to the amoebae *D. discoideum* and *A. castellanii* and that these differences could, in part, be ascribed to different genetic groups.

The basic level of virulence, counted as amoeba necessary for plaque formation, was generally higher for *A. castellanii*, even for the non-virulent isolates. The mean amoeba number of non-virulent strains for *A. castellanii* was about 194 amoebae and about 77 amoebae for *D. discoideum*. This could be due to the different growth temperatures. The optimum growth temperature for *A. castellanii* is 30°C, which represents also the optimum temperature for *S. maltophilia* growth. In contrast the optimum growth temperature for *D. discoideum* is 23°C, and results in slower growth of *S. maltophilia*, which might be a disadvantage.

One genetic subgroup (*gyrB* group E) revealed the largest number of virulent strains against both amoebae. First we chose only four isolates (NB12, GS1, SKK28 and c6) for testing virulence of group E. As all four isolates showed a high virulence potential, we tested more isolates from this group to confirm the overall emergence of virulent strains. In fact, not all isolates from group E were virulent, but indeed 8 from 12 tested isolates were characterized as highly virulent. Some *S. maltophilia* strains displayed a high virulence for *Acanthamoeba* or *Dictyostelium*, exclusively. Especially *gyrB* group D isolates were most virulent for *D. discoideum*, but not for *A. castellanii*. These discrepancies could not entirely be explained by different growth at different temperatures. We hypothesized that some bacterial strains showed temperature dependent differences in their protein expression profiles, which would somehow alter the interaction between bacteria and amoeba. In a single shot we conducted the experiment at 22.5°C with *Acanthamoeba*, the three strains from group D and strain SKK35 as positive control. Bacteria and amoebae grew slower at this temperature, and virulence could not be clearly differentiated until 5 days passed. but then it became clearly evident that group D strains did not show any virulence to *A. castellanii*, while the control strain SKK35 was highly virulent. This clearly showed that temperature dependence in virulence could be excluded. Another possibility is that *Dictyostelium* has some other defense mechanisms towards bacterial pathogens than *A. castellanii*.

An interesting outcome is that group A, with the highest number of respiratory tract isolates, exhibits no virulence at all in this model. These findings are contrary to the findings of Pompilio *et al.* [63], who demonstrated the development of an *S. maltophilia* CF-phenotype, but also showed that virulence of CF and non-CF strains in a murine lung infection model was at an equal level. Hence, we assume that different mechanisms would cause pathogenicity in respiratory tract infections in mice and in amoeba.

A major question for the interaction between *S. maltophilia* and amoebae is the pathogenicity mechanism itself. So far no intracellular growth of *S. maltophilia* in amoeba has been observed. Thus, the assumption arises that some extracellular compounds could act as cytotoxins. For *P. aeruginosa* it is known that a type-III-secretion system mediates toxicity to amoeba cells, for *Vibrio cholerae* virulence is mediated by a type-VI-secretion system. None of the fully sequenced and previously described *Stenotrophomonas* strains is known to have a type-III- or type-VI-secretion system. It would be either possible that the virulence mechanism of *S. maltophilia* is different and based on only extracellular compounds without intracellular invasion, or that only the strains virulent to the amoebae have these types of secretion systems. In this context, it has to be mentioned that the two fully sequenced strains K279a and R551-3, and the draft genome strain SKA14 are all non-virulent for both amoeba in this model.

Despite all previous successful applications of the amoeba virulence model, some deficiencies were revealed when *S. maltophilia* virulence became elucidated. Not all clinical *S. maltophilia* strains, known to have caused an infection in a human host, showed virulence in the amoeba model. Strain K279a, which was isolated from a blood culture of a cancer patient and was later characterized as virulent in a nematode model with *C. elegans*, showed no virulence towards the amoebae. The discrepancy of bacterial virulence for macrophage-like cells compared to *C. elegans* has previously been reviewed [64]. Nevertheless, this model gave us insight in the different pathogenicity properties of *S. maltophilia* strains against amoeba, which probably could be used for macrophages.

## Supporting Information

Table S1 List of *S. maltophilia* stains.
(DOC)
