# Supplementary material for: Genotyping of Environmental and Clinical Stenotrophomonas maltophilia Isolates and their Pathogenic Potential
Source: PLoS One. 2011 Nov 15;6(11):e27615. doi: 10.1371/journal.pone.0027615 (PMC3216982; doi:10.1371/journal.pone.0027615)
Supplement: Table S1 — List of S. maltophilia stains. (DOC) [file pone.0027615.s001.doc]

**Table S1**: List of *S. maltophilia* stains.

| **Strain no.** | **Analysis Method** | **Source** | **rep-PCR group** | ***gyrB* sequence group** | **Reference Group** | **Reference** | **EMBL no.** |
| --- | --- | --- | --- | --- | --- | --- | --- |
| DSM 50170 | 16S rRNA, *gyrB*, Rep-PCR | Oropharyngeal region of patient with cancer | 4 | A | 6 | Palleroni and Bradbury 1993 | FN395100 |
| **SKK1** | *gyrB*, Rep-PCR, virulence | Wound swab | NG | NG |  |  | FN395089 |
| **SKK2** | Rep-PCR | Urine | 7 |  |  |  |  |
| **SKK3** | *gyrB*, Rep-PCR, virulence | Respiratory tract | 7 | A |  |  | FN395081 |
| **SKK4** | Rep-PCR | Respiratory tract | 7 |  |  |  | FN395119 |
| **SKK5** | *gyrB*, Rep-PCR, virulence | Respiratory tract | 2 | G |  |  | FN395146 |
| **SKK6** | Rep-PCR | Vein catheter | 4 | E |  |  | FR847955 |
| **SKK9** | *gyrB*, Rep-PCR | Urine | NG | B |  |  | FN395085 |
| **SKK10** | Rep-PCR | Wound swab | 3 |  |  |  |  |
| **SKK11** | Rep-PCR | Human blood culture | 9 |  |  |  |  |
| **SKK12** | *gyrB*, Rep-PCR, virulence | Wound swab | NG | NG |  |  | FN395118 |
| **SKK13** | Rep-PCR, virulence | Urine | 4 |  |  |  | FR847960 |
| **SKK14** | Rep-PCR | Respiratory tract | NG |  |  |  |  |
| **SKK16** | Rep-PCR | Respiratory tract | 3 |  |  |  |  |
| **SKK17** | Rep-PCR | Urine | 3 |  |  |  |  |
| **SKK18** | Rep-PCR | Wound swab | 7 |  |  |  |  |
| **SKK19** | Rep-PCR | Respiratory tract | 7 |  |  |  |  |
| **SKK20** | Rep-PCR, *gyrB*, virulence | Respiratory tract | 4 |  |  |  | FR847956 |
| **SKK21** | *gyrB*, Rep-PCR | Respiratory tract | 7 | A |  |  | FN395120 |
| **SKK22** | Rep-PCR | Respiratory tract | 7 |  |  |  |  |
| **SKK23** | Rep-PCR | Wound swab | 6 |  |  |  |  |
| **SKK25** | Rep-PCR | Urine | 4 |  |  |  | FR847953 |
| **SKK27** | *gyrB*, Rep-PCR | Cervix swab | 3 | B |  |  | FN395147 |
| **SKK28** | *gyrB*, Rep-PCR, virulence | Urine | 4 | E |  |  | FN395111 |
| **SKK29** | Rep-PCR | Respiratory tract | 3 |  |  |  |  |
| **SKK30** | Rep-PCR | Urine | 2 |  |  |  |  |
| **SKK31** | Rep-PCR, *gyrB*, virulence | Wound swab | 4 |  |  |  | FR847961 |
| **SKK32** | Rep-PCR | Respiratory tract | 3 |  |  |  |  |
| **SKK33** | *gyrB*, Rep-PCR | Respiratory tract | 8 | A |  |  | FN395103 |
| **SKK34** | Rep-PCR | Urine | 10 |  |  |  |  |
| **SKK35** | *gyrB*, Rep-PCR, virulence | Ulcer swab | 3 | NG |  |  | FN395114 |
| **SKK36** | Rep-PCR | Wound swab | 10 |  |  |  |  |
| **SKK37** | Rep-PCR | Respiratory tract | 3 |  |  |  |  |
| **SKK38** | *gyrB*, Rep-PCR, virulence | Urine | 10 | D |  |  | FN395156 |
| **SKK39** | Rep-PCR | Contact lens | 5 |  |  |  |  |
| **SKK40** | Rep-PCR | Respiratory tract | NG |  |  |  |  |
| **SKK41** | Rep-PCR | Wound swab | NG |  |  |  |  |
| **SKK42** | Rep-PCR | Respiratory tract | 3 |  |  |  |  |
| **SKK44** | Rep-PCR | Conjunctiva swab | 3 |  |  |  |  |
| **SKK45** | Rep-PCR | Respiratory tract | 3 |  |  |  |  |
| **SKK46** | Rep-PCR | Ulcer swab | 7 |  |  |  |  |
| **SKK47** | Rep-PCR | Ulcer swab | NG |  |  |  |  |
| **SKK49** | Rep-PCR | Respiratory tract | 7 |  |  |  |  |
| **SKK50** | Rep-PCR | Respiratory tract | 8 |  |  |  |  |
| **SKK51** | Rep-PCR | Respiratory tract | 8 |  |  |  |  |
| **SKK52** | Rep-PCR | Respiratory tract | 7 |  |  |  |  |
| **SKK53** | *gyrB*, Rep-PCR | Respiratory tract | 12 | I |  |  | FN395144 |
| **SKK54** | Rep-PCR | Wound swab | 7 |  |  |  |  |
| **SKK55** | *gyrB*, Rep-PCR, virulence | Respiratory tract | 6 | C |  |  | FN395145 |
| **SKK56** | Rep-PCR | Wound swab | 3 |  |  |  |  |
| K279a | *gyrB* | blood of a cancer patient |  | A | 6 |  |  |
| 673 | *gyrB*, Rep-PCR | Respiratory tract | 7 | A | 6 |  |  |
| 674 | *gyrB*, Rep-PCR | Respiratory tract | 7 | A | 6 |  |  |
|  |  |  |  |  |  |  |  |
| 675 | Rep-PCR | Respiratory tract | 7 |  | 6 | Kaiser *et al.* 2008 |  |
| 676 | Rep-PCR | Respiratory tract | 7 |  | 6 | Kaiser *et al.* 2008 | FR847959 |
| 677 | *gyrB*, Rep-PCR | Respiratory tract | 7 | A | 6 | Kaiser *et al.* 2008 | FN395083 |
| 678 | *gyrB*, Rep-PCR | Respiratory tract | 12 | I | A | Kaiser *et al.* 2008 | FN395091 |
| 680 | Rep-PCR | Respiratory tract | 7 |  | 6 | Kaiser *et al.* 2008 |  |
| 681 | *gyrB*, Rep-PCR | Respiratory tract | 7 | A | 6 | Kaiser *et al.* 2008 | FN395121 |
| 682 | *gyrB*, Rep-PCR | Respiratory tract | 9 | F | 4 | Kaiser *et al.* 2008 | FN395149 |
| 683 | Rep-PCR | Respiratory tract | 5 |  | 2 | Kaiser *et al.* 2008 |  |
| 685 | Rep-PCR | Respiratory tract | NG |  | A | Kaiser *et al.* 2008 |  |
| 686 | Rep-PCR | Respiratory tract | NG |  | 3 | Kaiser *et al.* 2008 |  |
| c1 | Rep-PCR | Human | 7 |  |  | Minkwitz and Berg, 2001 |  |
| c2 | Rep-PCR | Human | NG |  |  | Minkwitz and Berg, 2001 |  |
| c3 | Rep-PCR | Human | 9 |  |  | Minkwitz and Berg, 2001 |  |
| c4 | Rep-PCR | Human | 2 |  |  | Minkwitz and Berg, 2001 |  |
| c5 | 16S rRNA, *gyrB*, Rep-PCR | Human | 2 | G |  | Minkwitz and Berg, 2001 | FN395157 |
| c6 | 16S rRNA, *gyrB*, Rep-PCR | Human | 4 | E |  | Minkwitz and Berg, 2001 | FN395130 |
| c7 | Rep-PCR | Human | 4 |  |  | Minkwitz and Berg, 2001 | FR847954 |
| c8 | Rep-PCR | Human | 6 |  |  | Minkwitz and Berg, 2001 |  |
| c9 | Rep-PCR | Human | 4 |  |  | Minkwitz and Berg, 2001 | FR847958 |
| c10 | *gyrB*, Rep-PCR | Human | 2 | G |  | Minkwitz and Berg, 2001 | FN395158 |
| c11 | *gyrB*, Rep-PCR | Human | 6 | C |  | Minkwitz and Berg, 2001 | FN395150 |
| c12 | Rep-PCR | Human | 6 |  |  | Minkwitz and Berg, 2001 |  |
| c13 | Rep-PCR | Human | NG |  |  | Minkwitz and Berg, 2001 |  |
| c14 | Rep-PCR | Human | NG |  |  | Minkwitz and Berg, 2001 |  |
| c15 | Rep-PCR | Human | 5 |  |  | Minkwitz and Berg, 2001 |  |
| c16 | *gyrB*, Rep-PCR | Human | 3 | NG |  | Minkwitz and Berg, 2001 | FN395151 |
| c17 | Rep-PCR | Human | 4 |  |  | Minkwitz and Berg, 2001 | FR847957 |
| c18 | Rep-PCR | Human | 9 |  |  | Minkwitz and Berg, 2001 |  |
| c19 | Rep-PCR | Human | 10 |  |  | Minkwitz and Berg, 2001 |  |
| c20 | 16S rRNA, *gyrB*, Rep-PCR | Human | 3 | NG |  | Minkwitz and Berg, 2001 | FN395129 |
| c21 | Rep-PCR | Human | 7 |  |  | Minkwitz and Berg, 2001 |  |
| c22 | *gyrB*, Rep-PCR | Human | 9 | F |  | Minkwitz and Berg, 2001 | FN395152 |
| c23 | Rep-PCR | Human | 3 |  |  | Minkwitz and Berg, 2001 |  |
| c24 | Rep-PCR | Human | NG |  |  | Minkwitz and Berg, 2001 |  |
| c25 | Rep-PCR | Human | 4 |  |  | Minkwitz and Berg, 2001 |  |
| LMG 10853 | 16S rRNA, *gyrB*, Rep-PCR | Sputum | NG | C | 3 | Hauben *et. al* 1999 | FN395164 |
| LMG 10873 | 16S rRNA, *gyrB*, Rep-PCR | Case of conjunctivitis | 10 | D | 7 | Hauben *et. al* 1999 | FN395160 |
| LMG 10874 | 16S rRNA, *gyrB*, Rep-PCR | Human blood culture | 9 | F | 4 | Hauben *et. al* 1999 | FN395162 |
| LMG 10991 | 16S rRNA, Rep-PCR | Leg, pus | NG |  | 2 | Hauben *et. al* 1999 |  |
| LMG 10996 | *gyrB*, Rep-PCR | Leg, ulcer | NG | B | 2 | Hauben *et. al* 1999 | FN395166 |
| LMG 11114 | 16S rRNA, *gyrB*, Rep-PCR | Human blood culture | 2 | G | 1 | Hauben *et. al* 1999 | FN395165 |
| LMG 10879 | 16S rRNA, *gyrB*, Rep-PCR | Rice paddy | 11 | J | 9 | Hauben *et. al* 1999 | FN395161 |
| e1 | 16S rRNA, *gyrB*, Rep-PCR | brackish lagoon | NG | NG |  | Minkwitz and Berg, 2001 | FN395123 |
| e2 | Rep-PCR | brackish lagoon | NG |  |  | Minkwitz and Berg, 2001 |  |
| e4 | *gyrB*, Rep-PCR | rhizosphere of oilseed rape | 11 | J | E2 | Minkwitz and Berg, 2001 | FN395153 |
| e7 | Rep-PCR | rhizosphere of oilseed rape | NG |  |  | Minkwitz and Berg, 2001 |  |
| e8 | Rep-PCR | rhizosphere of oilseed rape | 11 |  | E2 | Minkwitz and Berg, 2001 |  |
| e13 | 16S rRNA, *gyrB*, Rep-PCR | rhizosphere, oilseed rape | 7 | A |  | Minkwitz and Berg, 2001 | FN395124 |
| e19 | 16S rRNA, *gyrB*, Rep-PCR | geocaulosphere potato | 11 | J | E2 | Minkwitz and Berg, 2001 | FN395127 |
| e20 | 16S rRNA, *gyrB*, Rep-PCR | rhizosphere, potato | 11 | J | E2 | Minkwitz and Berg, 2001 | FN395131 |
| e21 | 16S rRNA, *gyrB*, Rep-PCR | sewage treatment plant | 9 | F |  | Minkwitz and Berg, 2001 | FN395126 |
| e22 | 16S rRNA, *gyrB*, Rep-PCR | sewage treatment plant | 10 | D |  | Minkwitz and Berg, 2001 | FN395154 |
| e23 | 16S rRNA, *gyrB*, Rep-PCR | eye care solution | 7 | A |  | Minkwitz 2001 | FN395168 |
| **GR3** | Rep-PCR | activated sludge | 1 |  |  |  |  |
| **GR4** | Rep-PCR | activated sludge | 1 |  |  |  |  |
| **GR5** | Rep-PCR | activated sludge | NG |  |  |  |  |
| **GR6** | Rep-PCR | activated sludge | 1 |  |  |  |  |
| **GS1** | *gyrB*, Rep-PCR | activated sludge | 4 | E |  |  | FN395090 |
| **GS2** | *gyrB*, Rep-PCR | activated sludge | 7 | A |  |  | FN395086 |
| **GS3** | *gyrB*, Rep-PCR | activated sludge | 7 | A |  |  | FN395107 |
| **GS5** | *gyrB*, Rep-PCR | activated sludge | NG | B |  |  | FN395110 |
| **GS6** | Rep-PCR | activated sludge | 3 |  |  |  |  |
| **GS7** | Rep-PCR | activated sludge | 7 |  |  |  |  |
| **GS8** | *gyrB*, Rep-PCR | activated sludge | 7 | A |  |  | FN395113 |
| **GS9** | Rep-PCR | activated sludge | NG |  |  |  |  |
| **KS3** | *gyrB*, Rep-PCR | activated sludge | NG | B |  |  | FN395109 |
| **KS8** | Rep-PCR | activated sludge | NG |  |  |  |  |
| **KS13** | *gyrB*, Rep-PCR | activated sludge | 11 | J |  |  | FN395132 |
| **KS14** | *gyrB*, Rep-PCR | activated sludge | NG | B |  |  | FN395094 |
| **KA24** | Rep-PCR | activated sludge | 3 |  |  |  |  |
| **KA41** | Rep-PCR | activated sludge | NG |  |  |  |  |
| **KA42** | Rep-PCR | activated sludge | 7 |  |  |  |  |
| **HA1** | *gyrB*, Rep-PCR | sewage plant effluent | 1 | H |  |  | FN395141 |
| **HA14** | Rep-PCR | sewage plant effluent | 1 | H |  |  |  |
| **HA22** | *gyrB*, Rep-PCR | sewage plant effluent | 11 | J |  |  | FN395135 |
| **NA16** | *gyrB*, Rep-PCR | sewage plant effluent | 1 | H |  |  | FN395138 |
| **NA18** | *gyrB*, Rep-PCR | sewage plant effluent | NG | NG |  |  | FN395136 |
| **NA20** | *gyrB*, Rep-PCR | sewage plant effluent | NG | NG |  |  | FN395143 |
| **NB2** | Rep-PCR | activated sludge | 1 |  |  |  |  |
| **NB12** | *gyrB*, Rep-PCR | activated sludge | 4 | E |  |  | FN395167 |
| **RA8** | *gyrB*, Rep-PCR | sewage plant effluent | 1 | H |  |  | FN395142 |
| **RA9** | *gyrB*, Rep-PCR | sewage plant effluent | 1 | H |  |  | FN395139 |
| **RA19** | *gyrB*, Rep-PCR | sewage plant effluent | 1 | H |  |  | FN395140 |
| **RB16** | *gyrB*, Rep-PCR | activated sludge | 7 | A |  |  | FN395137 |
| **B4** | *gyrB* | activated sludge |  | NG |  |  | FN395092 |
| **B6** | *gyrB* | activated sludge |  | B |  |  | FN395093 |
| **F4** | *gyrB*, Rep-PCR | activated sludge | 1 | H |  |  | FN395080 |
| **F11** | *gyrB*, Rep-PCR | activated sludge | 3 | H |  |  | FN395084 |
| **X434** | *gyrB*, Rep-PCR | water | 1 | H |  |  | FN395082 |
| **X743** | *gyrB*, Rep-PCR | water | 12 | I |  |  | FN395108 |
| **X968** | *gyrB*, Rep-PCR | water | 9 | F |  |  | FN395155 |
| R551-3 | 16S rRNA, *gyrB*, Rep-PCR | plant tissue, *Populus trichocarpa* | 1 | H | 5 | Taghavi *et al.* 2009 | CP 001111 |
| SKA14 | *gyrB*, Rep-PCR | marine | 3 | NG |  | Hagström *et al.* 2000 | FN395134 |
| NS1-5 | Rep-PCR | environment | 1 |  |  | Minkwitz & Berg 2001 |  |
| NS2-6 | Rep-PCR | environment | 1 |  |  | Minkwitz & Berg, 2001 |  |
| NS68 | Rep-PCR | environment | NG |  |  | Minkwitz & Berg, 2001 |  |
| NS296 | Rep-PCR | environment | 11 |  |  | Minkwitz & Berg, 2001 |  |
| **1.4** | Rep-PCR | freshwater sediment | 7 |  |  |  |  |
| **4.1** | *gyrB*, Rep-PCR | freshwater sediment | 7 | A |  |  | FN395079 |
| **4G** | Rep-PCR | freshwater sediment | 9 |  |  |  |  |
| **5.7** | *gyrB* | freshwater sediment |  | J |  |  | FN395105 |
| **5.8** | *gyrB*, Rep-PCR | freshwater sediment | 11 | J |  |  | FN395115 |
| **5F17** | Rep-PCR | freshwater sediment | NG |  |  |  |  |
| **6.7** | *gyrB*, Rep-PCR | freshwater sediment | 11 | J |  |  | FN395087 |
| **6F2** | Rep-PCR | freshwater sediment | 3 |  |  |  |  |
| **7.4** | *gyrB*, Rep-PCR | freshwater sediment | 11 | J |  |  | FN395106 |
| **7.5** | *gyrB*, Rep-PCR | freshwater sediment | 11 | J |  |  | FN395133 |
| **7E** | *gyrB*, Rep-PCR | freshwater sediment | 11 | J |  |  | FN395122 |
| **7F** | *gyrB*, Rep-PCR | freshwater sediment | 11 | J |  |  | FN395112 |
| **8.1** | *gyrB*, Rep-PCR | freshwater sediment | 11 | J |  |  | FN395104 |
| **8.5** | *gyrB*, Rep-PCR | freshwater sediment | 11 | J |  |  | FN395095 |
| **8.6** | *gyrB*, Rep-PCR | freshwater sediment | 11 | J |  |  | FN395077 |
| **8E** | *gyrB*, Rep-PCR | freshwater sediment | 11 | J |  |  | FN395078 |
| **8F** | Rep-PCR | freshwater sediment | NG |  |  |  |  |
| **8a1** | Rep-PCR | freshwater sediment | NG |  |  |  |  |
| VUN 10010 | *gyrB* | PAH-contaminated soil, abandoned factory |  | D |  |  | AY642281 |

The source of strains, methods the isolates were examined with and the assigned genomic groups, including grouping of reference strains from previous applications are shown. Strains isolated within this study are highlighted with bold letters. Sequences of the *gyrB* genes are submitted at the EMBL Nucleotide Sequence Database.
